# Workforce outcomes among substance use peer supports: a scoping review of individual and organizational influences

**DOI:** 10.3389/fpubh.2024.1515264

**Published:** 2025-03-11

**Authors:** Justin S. Bell, Dennis P. Watson, Tina Griffin, Sierra Castedo de Martell, Emma Sophia Kay, Mary Hawk, Bradley Ray, Michelle Hudson

**Affiliations:** ^1^Chestnut Health Systems, Lighthouse Institute, Chicago, IL, United States; ^2^University Library, University of Illinois Chicago, Chicago, IL, United States; ^3^School of Nursing, The University of Alabama at Birmingham, Birmingham, AL, United States; ^4^School of Public Health, University of Pittsburgh, Pittsburgh, PA, United States; ^5^RTI International, Research Triangle Park, Durham, NC, United States

**Keywords:** peers, peer recovery support services, substance use disorder, workforce development, burnout

## Abstract

**Introduction:**

Peer recovery support services (PRSS), an expanding component in SUD treatment, are delivered by individuals with lived experience of substance use disorder (SUD). Despite the growing importance of these peers and the unique challenges they face in a developing health profession, limited research has focused on their workforce outcomes. This review aims to map the literature on (a) workforce outcomes among peers and (b) the individual and organizational contributors to these outcomes.

**Materials and methods:**

We conducted a scoping review of empirical literature from January 1, 1999 to January 26 2023 on APA PsycINFO®, Embase®, CINAHL®, Web of Science™, and Google Scholar. We also conducted a search of grey literature on institutional websites to locate additional articles. Search strategies targeted terms related to peers (e.g., peer specialist, people with lived experience), workforce outcomes (e.g., burnout, compassion fatigue), and organizational environments (e.g., workplace, volunteer). The review was preregistered with Open Science Framework (https://doi.org/10.17605/OSF.IO/C9YNR).

**Results:**

Of the 16,361 total articles retrieved, 20 were included after screening, consisting of 9 quantitative, 9 qualitative, and 2 mixed-methods studies. Cross-sectional survey was the most common study design (*n* = 9). Organizational factors, such as supervisory support and professional development opportunities, were linked to increased job satisfaction and retention while factors such as inadequate compensation and stigma were barriers to workforce sustainability. Individual challenges, including boundaries with clients and a lack of self-care, were associated with burnout and decreased job satisfaction.

**Conclusion:**

Results highlight challenges faced by peers in SUD services which limit their ability to sustain well-being and achieve career longevity. Research gaps include the need for longitudinal studies, a clearer understanding of work settings, and an exploration of mediating or moderating factors affecting workforce outcomes. Future efforts to foster a sustainable peer workforce should focus on improving peer workers’ well-being through organizational support, professional development, and targeted interventions based on occupational health theories.

## 1 Introduction

Peer recovery support services (PRSS) are delivered by individuals (“peers”) who share lived or living experience with the populations they serve, and they play an increasingly prominent role in substance use disorder (SUD) treatment. Over half of SUD treatment facilities offered PRSS as of (1), an expansion aligned with the continuing professionalization of peers since certification of these roles began in 2001 (2). These efforts have been supported by several reviews suggesting that when peers serve as coaches or conduct intervention delivery, their clients report decreased substance use, increased rates of abstinence-based recovery, strengthened treatment retention, improved provider-participant relationships, and increased treatment satisfaction (3–7). However, efforts to expand PRSS and research regarding these efforts have often neglected to consider the well-being of peers themselves. Perhaps because of this oversight, high rates of turnover and burnout are reported among peers (8–10), jeopardizing not only the health of the peers but also the sustainability of the related services. This oversight underscores the need to identify specific conditions that contribute to negative peer workforce outcomes that can be used to develop strategies that support their well-being.

The PRSS workforce includes both certified and non-certified individuals (employed and volunteer) who provide a wide spectrum of services spanning from harm reduction initiatives to recovery efforts focused on abstinence (11). Distinct from sponsorship positions in mutual aid groups or certified addiction counselors with lived experience, peer roles have been carved out in various settings specifically designed to leverage the unique insights and experiences of individuals who have navigated SUD recovery (4, 12). Peers work alongside people in recovery or using substances and can complement clinicians or serve as standalone support (7). Their roles typically include a mix of emotional (e.g., sharing recovery stories, crisis support), informational (e.g., connection to information and referrals to recovery support), affiliational (e.g., connections to community supports), and instrumental support [e.g., housing assistance, vocational support; Reif et al. (7, 12) and White (12)]. The emergence of peer work as a profession began in 1999 when Georgia became the first state to authorize peer support as a billable service within both mental and behavioral health care settings (13). Since then, at least 43 states have introduced reimbursement for peer services in their Medicaid legislation (14, 15), and 49 offer peer credentialing (1, 16).

Supporting the growth of the PRSS workforce requires understanding the unique challenges peers face in their roles. Managing the emotional demands of the peer role while maintaining their own recovery creates inherent tension for these individuals (17, 18). The scope of their role is obfuscated by a lack of standards regarding training and examination, with each state outlining its own, often dissimilar, requirements for certification (5, 13, 15). Additionally, peers may face isolation within the broader healthcare workforce due to their unique qualifications. Often considered “non-clinical” with some healthcare teams professionals questioning their legitimacy, peers can be excluded from decision-making or assigned to tasks that do not fully utilize their unique expertise (19–21). At worst, peers’ association with a stigmatized group may subject them to harsher forms of discrimination within the organizations they serve (22).

Despite the fact that PRSS are becoming more valuable in the SUD service workforce, there is a limited understanding of the specific work-related challenges that peers face. The existing literature tends to focus on the effectiveness of PRSS interventions but often overlooks the well-being of the peers themselves. The overall objectives of this scoping review are to describe the nature and extent of literature on (a) peers’ workforce outcomes and (b) individual and organizational level contributors to these outcomes.

## 2 Materials and methods

### 2.1 Study design

We conducted a scoping review comprising primary source studies and grey literature describing workforce-related outcomes among peers. The review design was generated according to guidelines provided by Arksey and O’Malley (23), Westphaln et al. (24), and Mak and Thomas (25). Results are reported according to Preferred Reporting Items for Systematic Reviews and Meta-Analyses extension for scoping reviews (PRISMA-ScR). The review was preregistered on August 22, 2023 in the Center for Open Science’s Open Science Framework repository (https://doi.org/10.17605/OSF.IO/C9YNR). A protocol for this article was previously published (26).

### 2.2 Eligibility criteria

Eligibility was restricted to peer-reviewed empirical studies describing the experiences of peers who held formal roles along the full spectrum of PRSS (from harm reduction to recovery supports). For this review, the term “peer” encompasses individuals in SUD recovery, with or without state or organizational certification, as well as people who currently use drugs (PWUD) and provide PRSS. We included studies that examined workforce outcomes for peers and analyzed factors that influence these outcomes. Drawing from prior reviews on healthcare workforce outcomes, we developed a targeted list of outcomes for our search strategy (27–29). Our review was limited to studies conducted in the United States and published after January 1, 1999, coinciding with the emergence of formal peer certification. We excluded research on peer sponsorship roles within mutual aid organizations, as these positions involve reciprocal support in non-supervised contexts (30). Additionally, we omitted studies related to peer support in areas unrelated to SUD recovery and harm reduction, such as peers who provide exclusively mental health, physical health support, or housing linkage. To ensure accuracy in data extraction, only studies published in English were considered. The inclusion and exclusion criteria are summarized in Table 1.

**Table 1:** Screening inclusionary and exclusionary criteria.

Inclusion	Exclusion
Peer-reviewed qualitative or quantitative empirical studies	Not published in English
United States-based	Only discusses peers who are in “sponsorship” positions within substance use mutual aid organizations or people with lived experience working in a professional position (e.g., administrator, addiction counselor, social worker, therapist)
Discusses peer recovery support services (PRSS) in substance use harm reduction, treatment, or recovery	Discusses peers who work outside the substance use and harm reduction fields (e.g., mental/physical health, etc.)
Discusses certified and uncertified peers who are employed or volunteer in positions as well as people who use drugs (PWUD) who serve as peers	Published before 1/1/1999
Discusses workforce outcomes	

List of inclusionary and exclusionary criteria for screening identified literature.

### 2.3 Search strategies

Terms and strings to conduct our literature searches were designed by an information specialist (TG) and targeted several key databases and libraries, including APA PsycINFO® (EBSCO), Embase® (EBSCO), CINAHL® (EBSCO), Web of Science™ (Clarivate), and Google Scholar. Search strategies combined Medical Subject Heading (MeSH) and free-text terms related to peers (e.g., peer, people with lived experience), workforce outcomes (e.g., burnout, compassion fatigue), and organizational environments (e.g., workplace, volunteer). The final review and extraction of citations was conducted on August 22, 2023. Results were exported to the bibliographic manager Zotero and duplicates were eliminated with Rayyan (31). A full list of search strings by database is included in Supplementary Table 1.

As an additional step, we hand-searched websites of U.S.-based organizations with well-known influence within the PRSS field, including (a) Recovery Research Institute, (b) Addiction Policy Forum, (c) Peer Recovery Center of Excellence, (d) Substance Abuse and Mental Health Services Administration (SAMHSA), (e) Faces and Voices of Recovery, (f) National Association of Peer Supporters, (g) Peer Support Services Technical Assistance Center, and (h) Peers for Progress. Articles regarding the peer workforce were reviewed to determine if any included references to peer-reviewed literature met our eligibility criteria.

### 2.4 Study selection

**Figure 1:**
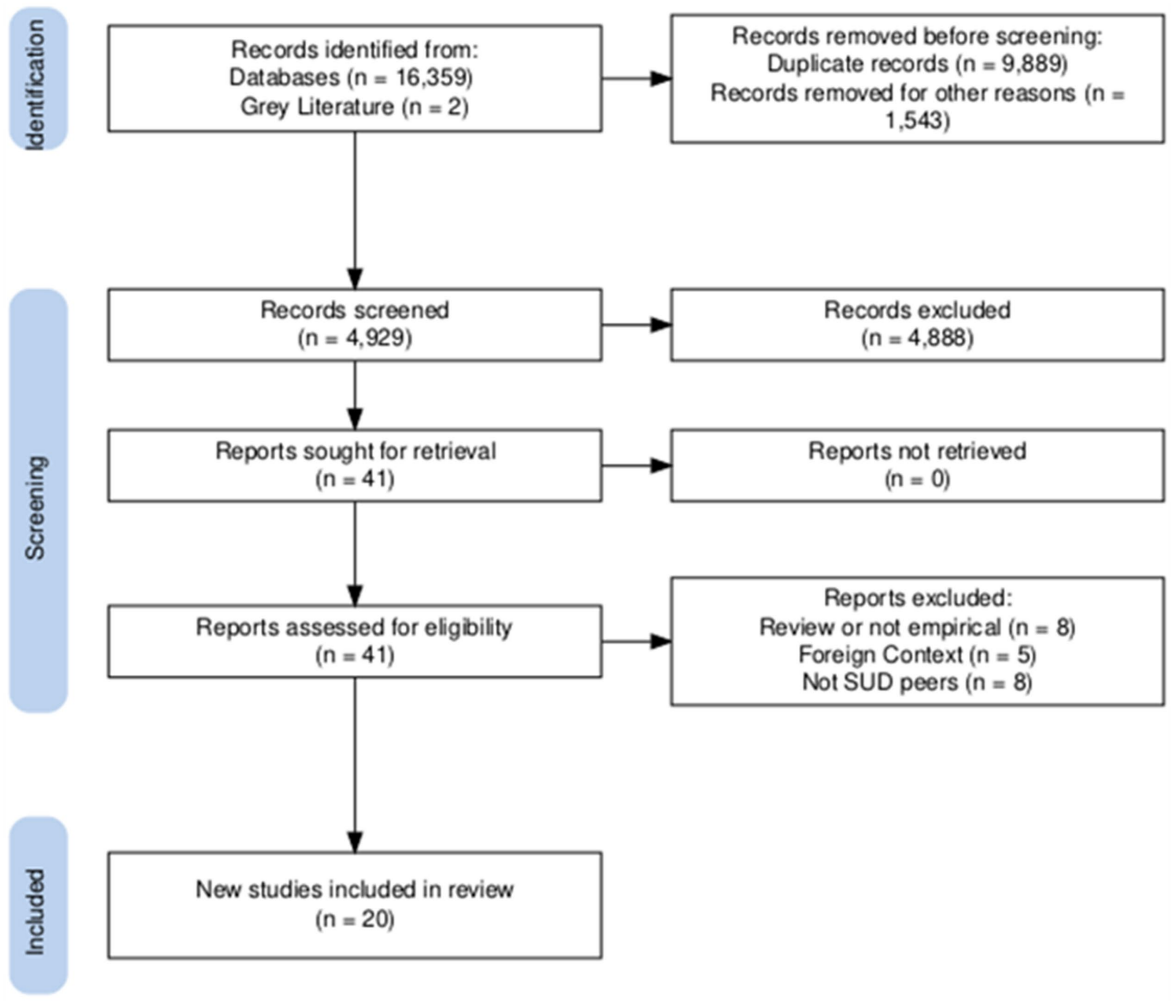
PRISMA flow diagram.

A total of 16,361 articles were identified through our initial literature searches. After removing duplicates and others for instantly disqualifying characteristics (i.e., published before 1999, not published in English), 4,927 articles remained. Two additional articles referenced in the grey literature were identified. Titles and abstracts of these remaining articles were screened by two authors (JSB, MH) and articles that met inclusion criteria underwent a second, full-text screening stage by the reviewers. Disagreements regarding eligibility at this stage were resolved by a third reviewer (DPW). A total of 20 articles were selected from this final stage. Figure 1 displays the screening and selection process followed, generated with the PRISMA online application (32).

### 2.5 Data extraction

Two authors (JSB, MH) extracted information from the articles with the qualitative analysis software MAXQDA, using a hybrid deductive-inductive approach to code *a priori* specified workforce outcomes and contributors to these outcomes from reviews of the healthcare and general workforce (27–29, 33, 34), as well as generate any new codes not in the *a priori* list (35, 36). The information coded included: (a) bibliographic information (publication type, year); (b) study location; (c) author thesis and research objectives; (d) sample size; (e) sample information, including peer definition and role type; (f) study methodology; and (g) context and workplace setting (e.g., rehabilitation center, recovery community organization, etc.). Primary outcomes were also recorded: (h) workforce outcomes (e.g., burnout, job satisfaction, vicarious trauma); (i) individual- and organizational-level contributors to workforce outcomes, as well as additional outcomes; and (j) author conclusions related to the support of peers. Coders piloted 5 articles to establish a Cohen’s Kappa statistic above 0.6 before dividing and independently coding the remaining articles.

### 2.6 Data synthesis

To guide and structure our results, contributing factors were classified by their facilitating or inhibiting relationship to our primary work-related outcomes (see Workforce Outcomes). Analysis was further supported by the generation of subthemes within codes using MAXQDA’s “AI Assist” functionality (37), contributing to our inductive process. For each primary code of our deductive coding scheme, 3–14 subcodes were generated by a large language model (GPT-4) that analyzed segments coded under the primary code (38). The authors reviewed subcodes for their validity and appropriateness, excluding irrelevant subcodes and suggesting further subcodes during discussion.

## 3 Results

### 3.1 Article characteristics

The characteristics and outcomes discussed for the 20 studies included in our analysis are described in Supplementary Table 2. Included are 9 quantitative, 9 qualitative, and 2 mixed methods studies. No grey literature was deemed eligible for final inclusion in our review. The most common study design was cross-sectional survey (*n* = 9) and most studies recruited their sample from peers in multiple settings, frequently via the distribution of surveys through certification bodies and professional groups (*n* = 16). When considering multiple setting samples, places where peers worked included recovery community centers, recovery housing, in-patient treatment centers, social service organizations, hospitals, crisis centers, drug court programs, collegiate recovery programs, and federal agencies. A discussion of identified themes is presented below, with subthemes ordered by frequency of discussion in the included articles.

### 3.2 Workforce outcomes

#### 3.2.1 Job satisfaction

Job satisfaction, or the extent to which peers felt contentment in their roles, was discussed in 13 articles (39–51). Job satisfaction was frequently discussed in the context of a sense of fulfillment or purpose received from the job. Peers enjoyed the ability to make a difference in the lives of others and a sense of purpose in leveraging their past experiences to help others (42, 47, 50, 51). Perhaps indicative of a higher sense of purpose, several quantitative studies reported high job satisfaction amongst peers, even when stress or burnout was an issue (45, 47, 51). Job satisfaction was found to be correlated with other outcomes, including role clarity and turnover intention (44, 47).

#### 3.2.2 Role clarity

Role clarity, or the degree of understanding of responsibilities, expectations, and boundaries associated with the peer role, was discussed in 9 articles (39, 40, 42, 43, 47, 52–55). Frequently, role confusion was described as plaguing the peer experience, contributing to discrepancies in peer work across contexts. Role confusion occurred when there was a lack of understanding and communication about the peers’ role among coworkers and supervisors, often leading to peers being treated like administrative or support staff rather than team members (39, 54, 55). Role drift, or task shifting, where peers perform tasks outside their scope, was also a common issue. Peers reported they were often asked to perform tasks disconnected from their primary work, leading to a sense of frustration when they felt their unique expertise was not being utilized (39, 41). Examples of tasks peers reported they were expected to perform outside of their scope included providing security for their facility, completing regulatory paperwork, and providing technology support to their clients (39, 43). The pandemic exacerbated these issues, as peers previously involved in advocacy, harm reduction work, and individual support were shifted to COVID-19-specific tasks, including assisting frontline workers and organizing client quarantine (41, 43). Role confusion was also shown to stem from the overlap between the roles of peers and other professionals like case managers and counselors (43).

#### 3.2.3 Burnout

Burnout, or the state of physical, emotional, and mental exhaustion experienced by peers due to prolonged and intense involvement in their work, was discussed in 7 articles (10, 39–41, 45, 48, 50). Articles described how the nature of peer roles put them at risk for exhaustion and burnout. The sometimes ambiguous scope of their role and natural inclination to connect with clients created conditions where peers felt overworked or emotionally drained (10, 39). In quantitative studies, approximately one- to two-thirds of respondents reported levels of exhaustion indicative of burnout (40, 45). These concerning levels were partially associated with structural and demographic factors. Peers who lived in rural areas, worked full-time, or had longer career tenure reported greater emotional exhaustion (10, 45). Peers also reported being less likely than non-peers to be offered remote work opportunities (i.e., work from home), contributing to burnout (41). However, one study found negative associations between burnout and demographic characteristics, where Black identity or increasing age were associated with lower burnout scores (40).

#### 3.2.4 Recovery benefits

The positive impact of the peer role on recovery was discussed as a workforce outcome in 6 articles (10, 39, 49–51, 54). Like their clients, peers reported receiving benefits as a result of their role including increased social support, self-reflection, self-esteem, and sense of purpose, which served to strengthen their personal recovery (10, 50, 51, 54). Peers were inspired when witnessing their clients’ growth and success in recovery but were also reminded of the importance of their personal recovery when clients struggled with recovery goals and returned to active substance use (10, 49).

#### 3.2.5 Return to uncontrolled use

Peers returning to uncontrolled use, also referred to as relapse or recurrence, was discussed in 4 articles (10, 49, 55, 56). Two articles discussed peers’ employment-related fears concerning this outcome were alleviated when organizations had specific procedures and planning in place in the event a peer returned to uncontrolled use (55, 56). Peers discussed uncertainty when organizations failed to plan for the event of returning to uncontrolled use including concern regarding their employment and belongings (39, 49). This was seen as a potentially alienating outcome for peers in their roles, as returning to use was reported as a source of shame and ostracism, even from fellow peers (39, 49).

#### 3.2.6 Retention and turnover

Peers’ retention in their roles and turnover were discussed in 3 articles (10, 45, 47). Despite challenging work conditions, peers were described as committed to their roles. One survey found peer turnover intention was low: the majority expected to remain in their roles long-term, with only a small percentage planning to leave within a year (45, 47). Qualitative reports confirmed peers wanted to stay in their roles but were compelled to leave in the face of workforce challenges (10). Turnover intention varied by experience and region, with higher intention to leave among peers with greater experience in the role and those in the Pacific region (45). Turnover intention was correlated with other workforce outcomes, including a positive correlation with burnout and negative correlation with job satisfaction (45, 47).

### 3.3 Organizational contributors to workforce outcomes

#### 3.3.1 Supervisor support and characteristics

Table 2 summarizes relationships and article counts for the organizational-level factors discussed. Supervisory support and characteristics of supervisors as contributors to optimal peer work outcomes were identified in 13 articles (39, 43–50, 52, 55–57). Ten of these articles discussed positive perceptions of supervisory support and the characteristics that generated these perceptions (43–49, 52, 55, 57). Supervisors were perceived as critical in fostering an organizational environment that supported self-care and professional development. Peers appreciated supervisors who encouraged taking time off, recognized peers for their accomplishments, and held conversations about self-care (46, 49, 55, 57). Qualities contributing to a perceived lack of supervisor support were discussed in five articles (39, 44, 49, 50, 56). Peers were not commonly supervised by other peers, and one study reported a desire for more supervisors with peer backgrounds who can be straightforward about the responsibilities of the role (39, 48). However, another study reported having a supervisor with a peer background was not significantly associated with increased job satisfaction (44). Three studies reported peers perceived a lack of supervisory support within their organizations, contributing to feelings of burnout (39, 49, 50).

**Table 2:** Organizational-level contributors to optimal peer workforce outcomes.

Organizational-level factor	Relation to optimal outcomes	Number of articles	Articles	Outcome(s) affected
Supervisory support and characteristics	Facilitator	10	1,3–11	Burnout, job satisfaction, retention, role clarity, return to uncontrolled use
	Barrier	5	2,5,10,12,13	
Opportunities for skill development and advancement	Facilitator	4	3,12–14	Retention
	Barrier	4	4,10,12,15	
Collaborative culture	Facilitator	5	1,5,11,13,16	Role clarity
	Barrier	5	2,5,7,11,16	
Role autonomy	Facilitator	5	1,2,8,10,11	Job satisfaction
	Barrier	2	8,17	
Support from other peers	Facilitator	4	1,3,8,10	Job satisfaction
	Barrier	2	2,17	
Interorganizational Relationships	Facilitator	4	1,3,8,14	Job satisfaction
	Barrier	1	12	
Training for Peers	Facilitator	4	8,11,15,18	Job satisfaction, role clarity, return to uncontrolled use
	Barrier	1	2	
Stigmatizing environments	Facilitator	0		Not discussed
	Barrier	4	3,8,11,15	
Payment and funding	Facilitator	0		Retention
	Barrier	4	2,8,12,14	
Employment benefits	Facilitator	4	1,2,10,12	Burnout, retention
	Barrier	1	14	
Recovery-oriented culture	Facilitator	3	2,9,12	Job satisfaction, return to uncontrolled use
	Barrier	1	2	

Organizational-level factors and their relationship to optimal workforce outcomes, ordered by most to least unique mentions in the literature.

##### 3.3.1.1 Associated outcomes

Perceptions of adequate supervisory support were discussed as a facilitator of increased job satisfaction, role clarity, reduced turnover intentions, reduced uncontrolled return to use, and decreased burnout (39, 44, 45, 47–50, 55).

#### 3.3.2 Opportunities for skill development and advancement

Professional development opportunities as a contributor to optimal peer work outcomes were identified in 7 articles (10, 43, 49–52, 56) with four discussing it as a facilitating factor (10, 43, 50, 56). Peers appreciated opportunities for professional development when available. Some settings had an established career ladder for peers to advance to supervisors (43). Other settings offered programming peers perceived enhanced professional skills, such as public speaking, networking, or expertise in specific topic areas such as nutrition or parenting (10, 50). In Texas and Pennsylvania, state policies introducing tiered systems of advancement and education played a role in peer advancement (56). Four articles discussed limited skill development and advancement opportunities as a barrier (49, 51, 52, 56), including a paucity of employers offering multistep career ladders or tuition reimbursement for degree programs (51, 52, 56).

##### 3.3.2.1 Associated outcomes

Professional development opportunities were discussed as a barrier to retention when not present. Peers reported not having long-term career goals and were not confident about chances for promotion, contributing to turnover issues (49, 52, 56).

#### 3.3.3 Collaborative culture

Collaborative organizational culture as a contributor to optimal peer work outcomes was identified in 7 articles (39, 44, 46, 50, 54, 55, 57). The importance and facilitation of peer and non-peer staff collaboration within organizations was discussed in 5 articles (44, 50, 54, 55, 57). Studies described the general acceptance of peers by their non-peer coworkers—especially the clinically trained staff—as helping peers feel appreciated in the settings they served (50, 54). These interprofessional relationships were discussed as extremely helpful in coordinating care. For example, peer coworkers are able to provide clients with information outside the peers’ area of expertise, generating buy-in for peer services and fostering inclusive workspaces (50, 57). Despite a perceived acceptance of peer work, 5 articles identified challenges to collaboration, primarily contributing to role confusion (39, 44, 46, 54, 55). In organizations where non-peers had not been educated concerning the peer role or where peers were isolated from their coworkers (“siloed”), non-peer staff tended to be confused by peer work and avoided engagement (39, 50, 54). A lack of familiarity with the relationship-building piece common to peer work led to this piece being devalued by non-peer staff. Tensions also increased when peers served in an advocacy role, such as speaking up to endorse destigmatizing language (39, 54).

##### 3.3.3.1 Associated outcomes

A lack of collaborative organizational culture was an associated barrier to role clarity (39, 50, 54, 55).

#### 3.3.4 Role autonomy

Role autonomy was identified as a contributor to optimal workforce outcomes in 6 articles (39, 41, 47, 49, 55, 57). Five articles discussed peer autonomy as a facilitating aspect of the role (39, 47, 49, 55, 57). Peers reported appreciating support from organizations that allowed flexible schedules and creativity in the delivery of services (39, 55, 57). The ability to express creativity in their roles was reported as important to peers and was promoted in community-based settings with relaxed organizational cultures (47). Two articles discussed limited autonomy as a barrier, influenced by certain work contexts and situations (41, 47). For example, in criminal-legal systems, autonomy was discussed as limited and peers felt less control over their roles (47). The sense of control also seemed to diminish during the COVID-19 pandemic. As services shifted to accommodate the crisis, peers’ roles were frequently shifted to tasks for which they were untrained or not originally hired to do, primarily through increased administrative tasks (e.g., scheduling, front desk support) as the logistics of one-on-one peer work was challenged and demand decreased (41).

##### 3.3.4.1 Associated outcomes

Although not a direct association, working in community settings that promoted role autonomy was discussed as a facilitator of job satisfaction, while settings that restricted autonomy were discussed as a barrier to satisfaction (47).

#### 3.3.5 Support from other peers

Support among peers was discussed as a contributor to optimal workforce outcomes in 6 articles (39, 41, 43, 47, 49, 57). Four articles discussed organizational involvement designed to increase support from peer coworkers as a facilitator of optimal work outcomes (43, 47, 49, 57). Organizations that employed multiple peers, created “peer-led” teams, and allowed time for peers to interact through meetings or social events seemed to foster connections between peers (43, 47, 49, 57). Two articles discussed perceived deficits in support from other peer workers (39, 41). Peers reported feeling isolated if they worked primarily with non-peer coworkers, expressing a desire for greater access to peer networks and mentorship (39). This sense of isolation increased as peer work shifted from in-person to virtual during COVID-19 (41).

##### 3.3.5.1 Associated outcomes

Support from colleagues was discussed as a positive predictor of job satisfaction (44).

#### 3.3.6 Interorganizational relationships

Opportunities to build relationships with external stakeholders were discussed in 5 articles (10, 43, 47, 56, 57). Four articles discussed inter-organizational relationships as a facilitator of optimal work outcomes (10, 43, 47, 57). Opportunities to interact with external stakeholders in the communities was reported to increase peer role engagement (10, 43, 47). These activities helped imbue a sense of purpose for peers by spreading knowledge of peer services to influential groups (43, 57). One document identified these relationships as a barrier to optimal work outcomes that increased the possibility of stigma toward peers, as external organizations were less likely to be knowledgeable about substance issues and recovery (56).

##### 3.3.6.1 Associated outcomes

Interorganizational relationships were discussed as facilitating job satisfaction through decreased isolation and increased engagement (47, 54).

#### 3.3.7 Training for peers

Training for peers was discussed as a contributor to optimal workforce outcomes in 5 articles (39, 47, 51, 53, 55). The benefits of providing adequate, in-person training for peers were discussed in 4 articles (47, 51, 53, 55) and training offered through organizations or certification bodies helped peers prepare for their roles (51). One study identified insufficient training in social determinants of health and co-occurring disorders as barriers to optimal outcomes (39). Training tended to focus solely on addiction support, while peer workers often work to address other daily client needs, including shelter, food, and transportation. Additional deficits were perceived in addressing co-occurring mental health concerns. Peers reported a lack of training can contribute to issues in their personal recovery, exacerbating negative recovery outcomes (39).

##### 3.3.7.1 Associated outcomes

Perceptions of sufficient training were associated with increased job satisfaction (53) with one-on-one training or in-person shadowing associated with greater role clarity over virtual training or self-study (47). The absence of training, especially covering substance use avoidance, was discussed as a facilitator of returning to uncontrolled use (39).

#### 3.3.8 Stigmatizing environment

Organizational environments with stigma toward peers and their pathways to recovery were noted in 4 articles, solely as a barrier to optimal workforce outcomes (43, 47, 51, 55). The use of medications for recovery, for example, was negatively associated with professional advancement (51). In one study, a significant proportion of peer respondents reported witnessing or personally experiencing discrimination from leadership or non-peer coworkers, most commonly through inequitable hiring or advancement practices (47). While peers also reported respondents felt stigma from fellow peers, stigma and discrimination was most typically received from non-peer staff (43, 47, 55).

##### 3.3.8.1 Associated outcomes

Specific associated workforce outcomes were not discussed.

#### 3.3.9 Payment and funding

Funding or compensation for peers was discussed in 4 articles, solely as a barrier to optimal work outcomes (10, 39, 47, 56). Common models for paying peers included “fee-for-service” or “block grant funding.” The former was perceived as a barrier as only certain services could be reimbursed by insurance. The latter had more flexibility but was perceived as less stable, with peers losing their funding after the grant ended (39). Peers reported their compensation was often not a satisfactory rate or was significantly lower than their non-peer coworkers (39, 47, 56).

##### 3.3.9.1 Associated outcomes

Inadequate and unstable compensation was discussed as a barrier to retention. Without adequate compensation, peers were forced to leave and find other work despite their desire to stay in the profession (10).

#### 3.3.10 Employment benefits

Employment benefits as a contributor to optimal work outcomes were identified in 4 articles (39, 49, 56, 57). Peers appreciated organizations providing adequate time off and resources during illness, stress, or difficulty maintaining their personal recovery (49, 56, 57). Peers also utilized employee assistance programs to access counseling to maintain their mental health (49). One document discussed perceived deficits in benefits (10). Focus group data collected from peers found that some organizations did not provide health insurance to their peer workforce and time-off requested by peers was scrutinized (10).

##### 3.3.10.1 Associated outcomes

Providing adequate time off for peer employees was discussed as a means of reducing burnout and supporting retention.

#### 3.3.11 Recovery-oriented culture

Strategies that facilitated an organizational emphasis on peers’ recovery and the impact on optimal workforce outcomes were discussed in 3 articles (39, 48, 56). Protecting time for peers to attend mutual aid meetings and providing training on the avoidance of returning to uncontrolled use both reflected an organizational culture that valued peers’ recovery (39, 48, 56). One document discussed policies that served as a barrier to peers’ recovery (39). Peers expressed frustration when protected time for meeting attendance was not provided, and their personal recovery routine was sidelined in favor of longer working hours, potentially contributing to an increased chance of returning to use (39).

##### 3.3.11.1 Associated outcomes

Recovery-oriented organizational culture was discussed as a positive predictor of job satisfaction and discussed as a barrier to returning to uncontrolled use (48, 56).

### 3.4 Individual contributors to workforce outcomes

#### 3.4.1 Maintaining boundaries with clients

Table 3 summarizes relationships and article counts for the individual-level factors discussed. Maintaining professional boundaries with clients was discussed as impacting workforce outcomes in 6 articles (39, 41, 42, 50, 56, 57). Two articles discussed the skill of maintaining boundaries as a critical component of the peer role (56, 57). This skill could be emphasized in training programs and employed by peers to maintain a positive work-life balance. Four articles discussed the challenges of maintaining these boundaries within the context of peer duties (39, 41, 42, 50). Peer roles tend to emphasize lived experience and imply an inherent emotional connection between peer and client. The strength of this empathy can also be a challenge when, struggling to discern between their personal and professional roles, peers were required to disengage with clients as a coping strategy (39, 42). Boundaries that were not properly maintained threatened peers’ recovery and was reported as more challenging to navigate when peers were experiencing symptoms of burnout (41, 42).

**Table 3:** Individual-level contributors to optimal peer workforce outcomes.

Individual-level factor	How discussed	Number of articles	Articles	Outcome(s) affected
Maintaining boundaries with clients	Facilitator	2	1,12	Burnout, return to uncontrolled use
	Barrier	4	2,13,17,19	
Engaging in self-care maintenance	Facilitator	3	1,2,10	Burnout
	Barrier	4	1,2,10,14	
External support networks	Facilitator	4	1,10,13,14	Not discussed
	Barrier	0		
Recovery-specific support	Facilitator	2	1,10	Not discussed
	Barrier	2	1,10	
Spirituality and religious Community	Facilitator	2	1,10	Not discussed
	Barrier	0		

Individual-level factors and their relationship to optimal workforce outcomes, ordered by most to least unique mentions in the literature.

##### 3.4.1.1 Associated outcomes

Difficulties maintaining boundaries with clients was discussed as resulting in a return to uncontrolled substance use and burnout (41, 42).

#### 3.4.2 Engaging in self-care maintenance

Self-care activities and their impact on optimal workforce outcomes were discussed in 4 articles (10, 39, 49, 57). Three discussed the importance of self-care in the peer role (39, 49, 57). Peers engaged in a variety of self-care activities to manage job stressors, including yoga, mindfulness, video games, and other strategies to decompress (49, 57). Traveling and taking time off also helped peers refresh their internal resources after long periods of work or crises (57). The same four articles discussed the absence of self-care as a barrier to work-life balance and a contributor to burnout. Some peers lacked self-care strategies or reported work interfered with their routines (39, 49). Peers also discussed times when they prioritized clients over their self-care, especially when clients were in crisis (10).

##### 3.4.2.1 Associated outcomes

Self-care was discussed as a strategy to avoid burnout (57).

#### 3.4.3 External support networks

External support networks (i.e., family, friends) were discussed as impacting optimal workforce outcomes in 4 articles, solely as a facilitator (10, 49, 50, 57). Peers reported family members and close friends played a crucial role in supporting their well-being and recovery. The ability to decompress and process the day’s challenges with trusted individuals was highlighted as a valuable self-care practice (10, 49, 57). Peers also highlighted moments where they could share success in their roles with family and friends, increasing a sense of pride in their work (50).

##### 3.4.3.1 Associated outcomes

Specific associated workforce outcomes were not discussed.

#### 3.4.4 Recovery-specific support

The impact of recovery networks and communities was discussed as impacting optimal workforce outcomes in 2 articles (49, 57). These articles emphasized the importance of peers having a personal support network. Recovery-specific support activities included attending mutual aid meetings and staying in contact with members of these groups (e.g., sponsors) (49, 57). Maintaining recovery support was discussed as an ethical principle for peers identifying as being in recovery, as they could not effectively support others without tending to their personal wellbeing (57). The challenge, however, was avoiding contact with clients (49, 57). Peers discussed feeling uncomfortable sharing during mutual aid meetings where current or former clients were also in attendance, sometimes attending groups in other cities to avoid contact or abstaining from meetings altogether.

##### 3.4.4.1 Associated outcomes

Specific associated workforce outcomes were not discussed.

#### 3.4.5 Spirituality and religious community

The impact of spirituality and religious community on workforce outcomes was discussed in 2 articles, solely as a facilitator (49, 57). Spiritual practices were considered essential to self-care, supporting recovery and work (57). Participating in religious or spiritual groups was discussed as a source of community. Religious groups could also provide areas for service and socializing outside of work, helping to grow peers’ support networks (49).

##### 3.4.5.1 Associated outcomes

Specific associated workforce outcomes were not discussed.

#### 3.4.6 Other factors

Two factors did not meet the cut-off to qualify as separate themes but could be important areas of future direction. Race/ethnicity was discussed as a barrier in one document, with non-White peers reporting lower average job satisfaction than White peers (46). Clinical mental health support was discussed as a source of support in one document, with peers listing counselors alongside family or friends as key members of their networks (49).

### 3.5 Summary of the results

The scoping review revealed several key workforce outcomes and organizational contributors affecting SUD peer supports. Job satisfaction was generally high among peers, often linked to feelings of purpose and fulfillment, though negatively impacted by burnout and role confusion. Role clarity was frequently compromised due to unclear expectations, role drift, and overlapping responsibilities with non-peer staff, which intensified during the COVID-19 pandemic. Burnout was prevalent, particularly among rural, full-time, and peers with longer career tenure, with some demographic factors (e.g., Black identity, older age) influencing its impact. Despite challenges, peers reported recovery benefits such as enhanced self-esteem and social support, although returning to uncontrolled use posed professional and emotional challenges, particularly in the absence of clear organizational policies. Retention and turnover rates were influenced by burnout, job satisfaction, and regional factors, with many peers expressing commitment to their roles despite systemic barriers. Organizational factors such as supervisory support, professional development opportunities, and collaborative cultures were critical in shaping workforce outcomes. Supportive supervision, skill-building initiatives, and positive interprofessional relationships improved job satisfaction and reduced turnover intentions. Conversely, limited autonomy, inadequate training, and stigmatizing environments hindered workforce engagement and satisfaction.

## 4 Discussion

Overall, the literature suggests that many peers are challenged in their roles by insufficient organizational support and inadequate professional competencies which interfere with their ability to maintain boundaries, manage stress, and sustain their personal recovery. Workforce conditions, including low wages and a lack of advancement opportunities, often prevent peer work from being a viable long-term career option and is likely to become a more significant issue as peers are further integrated into the treatment continuum (58). Despite these issues, peers report a strong desire to remain in their role and derive both personal and professional benefits from applying their lived experience to the service of people with substance use issues. The following discussion offers recommendations researchers, organizations, and peers can implement to address these findings, foster supportive environments for peer workers, and enhance peers’ overall well-being and effectiveness.

### 4.1 Implications for research

The scoping review highlights critical gaps in the literature on SUD peer workforce outcomes. Current research on this workforce remains underdeveloped, particularly regarding relationships between workforce-related outcomes and their contextual contributors. Most included studies utilized cross-sectional, survey-based designs which limit the ability to draw causal inferences regarding workforce outcomes. Additionally, there was limited exploration of potential mediating or moderating factors. Future research would benefit from employing mixed methods and multivariate analyses to examine these relationships more comprehensively and offer more profound insights into the mechanisms that drive workforce outcomes.

The number of studies that contextualized peer outcomes to the settings where they worked was limited. For example, role autonomy was more frequently reported in community-based settings while environments where peers were given strict oversight, such as those within the criminal-legal system, seemed to limit autonomy (47). Most eligible studies were large surveys of peers and, while the settings that comprised their sample were reported, the studies did not contextualize outcomes to the work environment. These gaps prevent a full understanding of the unique needs and challenges peer workers face in different settings and limits the ability to develop targeted workforce supports. Related to workplace, there is a need to understand how context might impact outcomes through its influence on peers’ ability to practice to the full extent of their professional role. For instance, could working in a setting that might prevent peers from linking clients to medications for opioid use disorder or from providing harm reduction resources (e.g., criminal justice settings) contribute to burnout? Other contextual considerations include peers’ demographic characteristics, including race and gender. Previous research has found certain demographic groups are underrepresented in existing pathways of advancement (59). Very few articles included in this review explored demographic characteristics related to workforce outcomes but the finding that non-White peers reported lower job satisfaction indicates a need for further exploration (46).

Longitudinal studies are notably absent from the existing literature. This is a major limitation, as workforce outcomes like job satisfaction, turnover intentions, and burnout may fluctuate over time due to changes in organizational structure, recovery status, and external stressors. For example, perceptions of supervisory support and professional development opportunities likely evolve as peer workers advance in their careers (60). Additionally, peer workers’ ongoing recovery journeys may affect their job performance and satisfaction. Aspects include the pathways to recovery in which peers engage and their relation to work, as studies tended to focus exclusively on 12-step recovery [e.g., Wohlert (49)]. Long-term studies could provide valuable data on how these factors influence career trajectories, retention, and well-being.

A critical next step is identifying the workforce outcomes that most matter to peer workers themselves (61, 62). This review identified several key outcomes, such as job satisfaction, role clarity, and burnout, all of which are closely linked to organizational factors like supervisor support and opportunities for professional development. However, few studies directly asked peer workers which aspects of their jobs they prioritize. Tailored research in this area could inform the development of interventions that address the unique concerns of peer workers. For instance, interventions which enhance peer networks, ensure adequate training, or provide pathways for career advancement could significantly improve retention and job satisfaction. While some challenges faced by peer workers, such as emotional exhaustion or the impact of insufficient resources, are common to other emotionally demanding professions (e.g., caregiving roles), certain factors are unique to peer work. These include navigating dual roles as both providers and individuals with lived experience, overcoming stigma associated with their own recovery journeys, and managing role-specific expectations that may differ from traditional behavioral health staff. Research informed by occupational health theories, such as the Job Demands-Resources or Conservation of Resources theories, could provide critical insights into how to balance the demands placed on peer workers with the resources available to them (63). Examining factors like workload, role clarity, access to professional development, and support from non-peer coworkers will be essential for understanding how these elements mitigate or exacerbate burnout. Effective leadership and supervision models that foster a supportive work environment, promote autonomy, and incorporate trauma-informed supervision practices are especially important for improving job satisfaction and retention. Using occupational theory in peer workforce research can thus guide the development of targeted interventions that promote sustainability in this workforce, ultimately improving retention and service quality.

### 4.2 Implications for organizations

Organizational strategies that contributed to peers’ positive experiences in their roles often reflected an overarching “organizational culture” or the shared values, beliefs, norms, and practices that shape the behavior, attitudes, and interactions of members within an organization (64). Establishing an organizational culture that supports peers includes policies that are helpful for their work and well-being, but also includes changing the norms and expectations about appropriate attitudes and behaviors toward peers held by non-peer staff. Normative influence is considered a more powerful way of guiding behavior within organizations, as underlying attitudes and motivation limit formal directives (65, 66). Organizational theories of cultural change stress a “process approach” whereby change is acknowledged as complex, chaotic, and shaped by the consequences of “resistance, political processes, negotiations, ambiguities, diverse interpretations, and misunderstandings” on the part of the members (67–70). Therefore, it is not the change itself but how the change is implemented that may be key to creating authentic, positive workspaces for peers. The best course of action is likely finding opportunities for peers and their non-peer counterparts to voice their opinions, provide feedback, and generate unique solutions within each organization. However, as a starting framework, three characteristics of a desired organizational culture were described in the literature: recovery-oriented, collaborative, and sustainable.

An organizational culture that is “recovery-oriented” includes the belief that peers are people in recovery first and foremost, with values of self-care and well-being reflected in organizational policies and supervisory practices. Peers reported that dedicated time for maintaining their recovery and self-care was crucial for avoiding burnout and return to uncontrolled use (39, 48, 56). Much of this cultural component was facilitated by supervisory staff, who are the most likely to hold conversations concerning self-care strategies and coordinate well-being-related benefits (i.e., time off). Organizations like the National Association of Peer Supporters (N.A.P.S.), the Peer Support Services Technical Assistance Center, and Peers for Progress have developed recommendations and guidelines for supervising peers and encourage ongoing communication between supervisors and peers regarding challenges in the role and flexibility to ensure well-being and professional development (71–73). Peers also discussed fears concerning a return to substance use, viewing their training as inadequate to prevent it and worrying about the stigma surrounding its occurrence and the consequences to their social support and professional status (39, 49). Organizations can work to address these fears by holding substance use (or relapse) prevention training for their peer staff, including information that helps reduce the stigma and uncertainty associated with returning to uncontrolled use. Additionally, organizations employing SUD peer workers can provide harm reduction resources, such as naloxone and safer use supplies, as more proactive measures. Fostering a non-punitive approach to substance use, including peer-led support groups, counseling services, and reintegration plans, can ensure that peers who return to use are supported rather than alienated within organizations.

“Collaborative” organizational culture includes a belief that peers are just as important as non-peers to the mission and success of the organization. Components include values of respect and teamwork among members and conditions that encourage partnerships between peers and non-peers. Several articles referred to the challenges of maintaining a collaborative culture within organizations, especially as peers’ work was siloed from other staff. Siloing contributed to a lack of knowledge from non-peers regarding the role of peers, which was especially harmful when clinical staff held significant influence over the organization’s culture or served as supervisory staff (39, 50, 54). Cultivating a collaborative organizational culture can involve promoting interaction between peer and non-peer staff through joint training sessions, team-building activities, and regular communication channels. As recommended by SAMHSA’s Peer Recovery Center of Excellence, including non-peer staff in the hiring and onboarding process can develop connections early on, and should be reinforced with education for these coworkers on the scope of the peer role (74). Additionally, interprofessional education techniques, including workshops and role-playing simulations, can help peers and non-peers learn about each other’s roles and collaborate effectively (75, 76).

Finally, organizations that promoted a culture of professional “sustainability” were associated with lower turnover and higher job satisfaction among peers (10, 43, 50, 56). Sustainability within an organization’s culture includes a belief that the peer role is a long-term career pathway, reflected in opportunities for advancing professional development. Notably, turnover was the most prevalent among peers with greater experience in the role, potentially contributing to a form of “brain drain” as those with expertise leave as they struggle to advance. Strategies to support retention should target these veteran peers, including promoting peers to supervisory positions and creating opportunities for professional advancement through career ladders (59). Sustainability also involves allowing for autonomy and creativity in the peer role. “Job crafting,” or the ability for an employee to actively modify or shape their own job roles and tasks to better align with their skills, interests, and values, is an important aspect of the peer role as they constantly draw upon their personal experiences to connect with and support others (47, 63). Finally, sustainability would be incomplete without the support of peers’ financial well-being. Our findings support a recent analysis showing average peer wages are consistently lower than the local living wage (77). Faces and Voices of Recovery, a national organization advocating for peer professional advancement, has offered various recommendations to support financial well-being, including the active monitoring of compensation by policymakers and payment reforms that increase reimbursement to cover the full scope of peer services, including provisions to compensate for time spent in self-care (78). Beyond supporting peers’ financial well-being, organizations should also consider how their funding for peers reflects their value within the organization. Peers who are subcontracted, rather than hired, may become peripheral to the organization’s culture and decision-making processes, limiting integration and collaboration with non-peers.

An important consideration in developing these organizational strategies is the ethical responsibility organizations bear when employing peers. Peer workers bring immense value through their lived experiences, but these same experiences can also make them vulnerable to unique challenges that must be addressed equitably and thoughtfully. Organizations must first assess their readiness to employ peers, ensuring that their workplace culture, infrastructure, and staff are prepared to integrate peers effectively. To address these responsibilities, SAMHSA (79) offers clear recommendations: define and clarify the peer role for all staff before hiring and with Human Resources, enhance the capacity to recruit and hire peers, promote a workplace culture that supports peer specialists, educate and support non-peer staff, establish effective supervisory practices, address job difficulties faced by peers, and promote universal designs for employee wellness, resilience, and self-care. Ethical considerations become even more complex when peers are former clients of the hiring organization. These situations can introduce potential conflicts, such as discomfort between peers and clinical staff or blurred boundaries when former clients work with current clients. Organizations can mitigate these challenges through clear policies on dual relationships, considering factors like the length of time since the peer was a client and existing relationships within the organization (79). By approaching peer employment with intentionality and ethical foresight, organizations can ensure that their practices uphold the well-being of both peers and the clients they serve.

### 4.3 Implications for peers

Peers’ personal behaviors—ranging from engagement with external support networks to self-care practices—also play a significant role in shaping their well-being in the workplace. Findings highlight actions peers can take in their daily lives to avoid the effects of stress in their roles. However, the benefits of these personal actions are limited when conditions placed upon them by organizations or policy limit their ability to achieve well-being. Emerging advocacy efforts are necessary to help uplift the voices of peers in conversations regarding their future.

Literature described several personal strategies that helped combat negative workforce outcomes such as burnout, returning to uncontrolled use, and job dissatisfaction. Peers discussed the importance of actively seeking and nurturing relationships with family, friends, and recovery communities that supported their well-being (10, 49, 50, 57). Maintaining well-being also included regular engagement in self-care as well as activities designed to rejuvenate and destress, including physical, creative, spiritual, and relaxation activities (39, 49, 57, 80). Self-care holds unique importance for peers given their dual responsibility to their own recovery and that of their clients. Social support and self-care activities can act as preventive maintenance for peers to avoid negative outcomes and ensure their clients receive the best support possible (61, 81). SAMHSA currently recommends a variety of individual and organizational-level interventions for addressing burnout in the behavioral workforce which have the prospective to be equally beneficial for peers (61).

Likely to be more productive, however, is the role of collective action among peers to ensure better workforce conditions. While not explicitly discussed in the literature featured in this review, professional groups and labor organizations are beginning to serve as protectors against suboptimal working conditions (82). National organizations, like N.A.P.S., are actively engaging with lawmakers to generate legislation to address many of the challenges featured in this review, including fair hiring practices, higher wages, and recognition of peers as a valid legitimate professional group (83). Peer service organizations should also be compelled to address the recommendations featured in this review. Professional groups in many states are beginning to assist in this effort. These groups can collectively organize peers to gain awareness by forming advocacy councils, holding conversations with peer providers, and voicing their opinions to local legislators [e.g., VERBI Software (37)]. These efforts mirror the success of professional groups to raise the status of other non-traditional workforces, including community health workers (CHWs) and certified nursing assistants [CNAs; Brady et al. (57) and Felton et al. (39)]. Ultimately, professional and advocacy groups can work “independently of employers, funders, and state agencies” to ensure peer voices guide policy and organizational planning (84, p. 12).

### 4.4 Limitations

Limitations are noted in this work. Our scoping review may have missed relevant articles, especially given the variability in definitions used to describe the peer workforce, as well as negative or positive work-related outcomes and their contributors. This scoping review may have missed relevant data from unpublished studies or reports, and very few instances of grey literature were gathered from our searches of the featured databases. Additionally, there was a gap between the search cut-off date and the final write-up of this review, during which newly published research may not have been captured. Although assessments of quality are not a requirement of scoping investigations (85), a lack of quality assessment may have led to the inclusion of lower-quality studies that limited the strength of the conclusions drawn. Augmenting our thematic analysis with large language models like GPT-4 exemplifies the potential for “human-AI synergy” to enhance data synthesis (86) but could have introduced bias into our results. The authors carefully considered the risks of AI usage (e.g., overreliance on frequently occurring words, inherited biases from training data) while also acknowledging prior studies demonstrating how AI can serve as a useful starting point for deeper analysis (87, 88). Ultimately, this synergy not only improved the efficiency of our thematic analysis but also contributed to richer insights in our review. Finally, it is noted that studies were limited to those published in English and settings within the United States, limiting the generalizability of the findings to other contexts or cultural settings.

## 5 Conclusion

The findings highlight significant gaps in the knowledge regarding the workforce experiences of peers providing SUD services. Key gaps include a lack of longitudinal studies, limited exploration of workplace and role types, and insufficient consideration of mediating and moderating factors that influence workforce outcomes. These gaps hinder a full understanding of the factors contributing to long-term retention, job satisfaction, and burnout among peer workers. While the knowledge is limited, however, it does provide a basis from which to begin identifying strategies for improving peers’ work situations when combined with general occupational health knowledge regarding factors that facilitate positive workforce outcomes. Such efforts are vital for ensuring the stability and resilience of peer workers as they play an increasingly vital role in SUD services.
