# Supplementary material for: Workforce outcomes among substance use peer supports: a scoping review of individual and organizational influences
Source: Front Public Health. 2025 Mar 11;12:1515264. doi: 10.3389/fpubh.2024.1515264 (PMC11935349; doi:10.3389/fpubh.2024.1515264)
Supplement: Supplementary file 2 [file Table_2.docx]

| **Supplemental Table 2. Characteristics of Included Studies** | | | | | | |
| --- | --- | --- | --- | --- | --- | --- |
| **Authors/ Year** | **Type of Publication** | **Sample Size** | **Study  Type** | **Study  Design** | **Context/ Setting** | **Outcomes Discussed** |
| Brady et al., 2022 | Research Article | 20 | Qualitative | Semi-structured  interviews | Multiple Settings (undefined) | Job Satisfaction |
| Felton et al., 2022 | Research Article | 2 (*n =* 9) | Qualitative | Focus groups | Multiple Settings (local SUD treatment agencies) | Burnout, Job Satisfaction, Recovery Benefits, Return to Uncontrolled Use, Role Clarity, Task Shifting |
| Tate et al., 2021 | Research Article | 9 (*n* = 63) | Qualitative | Survey and focus groups | Multiple Settings (undefined) | Burnout, Retention, Recovery Benefits |
| Pasman et al., 2022 | Research Article | 266 | Quantitative | Survey | Multiple Settings (substance use treatment or recovery programs, community mental health agencies, healthcare settings, criminal legal system, other) | Burnout, Role Clarity, Job Satisfaction |
| Unachukwu et al., 2023 | Research Article | 30 | Qualitative | Qualitative interviews | Multiple Settings (harm reduction settings, SUD treatment) | Burnout, Retention, Task Shifting |
| McCarthy et al., 2022 | Research Article | 17 | Qualitative | Semi-structured  interviews | Other (child welfare system program) | Job Satisfaction, Retention, Role Clarity, |
| Adams et al., 2022 | Research Article | 1280 | Quantitative | Survey | Multiple Settings (peer-run organization, peer-run mental health or social service agency, government agency or organization) | Job Satisfaction, Retention, Task Shifting |
| Lapidos et al., 2018 | Research Article | 319 | Quantitative | Survey | Multiple Settings (community mental health, clubhouse or drop-in center, and other, US Dept. of Veteran Affaris, forensic settings and health plans) | Role Clarity |
| Edwards, 2022 | Dissertation | 507 | Quantitative | Survey | Multiple Settings (hospitals, community-based organizations, residential settings, respite settings, and non-direct care service settings) | Job Satisfaction |
| Madaris, 2023 | Dissertation | 414 | Quantitative | Survey | Multiple Settings (Undefined) | Burnout, Job Satisfaction, Retention |
| Mowbray et al., 2021 | Research Article | 252 | Quantitative | Survey | Multiple Settings (attendees of training conference) | Job Satisfaction |
| Cronise et al., 2016 | Research Article | 597 | Quantitative | Survey | Multiple Settings (community and/or peer-run program settings, mental health and substance abuse treatment settings, residential settings and programs, pre-crisis or crisis settings, employment or educational settings, criminal justice settings, other) | Job Satisfaction, Retention, Role Clarity |
| Abraham et al., 2021 | Research Article | 117 | Quantitative | Survey | Multiple Settings (mental health center, inpatient psychiatric hospital, US Depart. of Veteran Affairs, drop-in center, crisis center, health department, substance abuse rehab, physical health rehab/assisted living, hospital, consumer led programs, other) | Burnout, Job Satisfaction, Role Clarity |
| Jenkins et al., 2017 | Research Article | 195 | Quantitative | Survey | Multiple Settings (Undefined) | Job Satisfaction, Role Clarity |
| Wohlert, 2014 | Dissertation | 10 | Qualitative | Semi-structured  interviews | Peer-Run Organizations | Job Satisfaction, Recovery Benefits, Retention, Return to Uncontrolled Use |
| Adams et al., 2023 | Research Article | 39 | Qualitative | Interviews | Multiple Settings (behavioral health, social service agencies) | Recovery Benefits, Retention, Role Clarity, Task Shifting |
| Chapman et al., 2018 | Research Article | 194 | Qualitative | Comparative case study | Multiple Settings (mental health clinics, detox and rehabilitation centers, crisis stabilization units, hospitals, peer-run respites, community centers, supportive housing, sobering houses) | Retention, Return to Uncontrolled Use |
| Dehart & Mason, 2022 | Research Article | 23 | Qualitative | Qualitative interviews | Criminal Justice Setting | Job Satisfaction |
| Hagaman et al., 2023 | Research Article | 565 | Mixed Methods | Focus groups and quantitative instrument | Multiple Settings  (community mental health, recovery community center, recovery residence, in-patient treatment, MAT clinic, social services organization, homeless shelter, justice system, drug court, hospital ED, church or faith-based organization, primary care, collegiate recovery program, child welfare agency, other) | Job Satisfaction, Recovery Benefits |
| Alberta et al., 2012 | Research Article | 23 | Mixed Methods | Observation and  quantitative instrument | Peer-Run Organizations | Return to Uncontrolled Use, Role |

*Note.* Details of articles included in the scoping review.
